# Usability of a blood-based HIV self-test kit in Lusaka province, Zambia: A cross-sectional analysis

**DOI:** 10.4102/ajlm.v14i1.2903

**Published:** 2025-12-20

**Authors:** Fales Z. Mwamba, Mwansa Mwape, Brenda C. Simfukwe, Nowella M. Musunga, Evans M. Mathebula, Geofrey Mupeta

**Affiliations:** 1 Department of Laboratory, Levy Mwanawasa University Teaching Hospital, Lusaka, Zambia; 2 Global Health Supply Chain - Procurement and Supply Management, Chemonics International, Lusaka, Zambia; 3 Abbott Rapid Diagnostics (Pty) Ltd, Pretoria, South Africa; 4 School of Health Systems and Public Health, Faculty of Health Sciences, University of Pretoria, Pretoria, South Africa; 5 Department of Pathology and Microbiology, School of Medicine and Health Sciences, Mulungushi University, Livingstone, Zambia

**Keywords:** CheckNOW™, HIV self-test, usability, awareness, user-friendliness

## Abstract

**Background:**

Despite progress toward the Joint United Nations Programme on HIV/AIDS (UNAIDS) 95-95-95 targets, Zambia faces persistent gaps in HIV testing coverage. The Ministry of Health implemented blood-based HIV self-testing (HIVST) to improve accessibility. This study evaluated the CheckNOW™ HIVST kit’s usability in Lusaka province health facilities.

**Objective:**

To determine usability, awareness and user-friendliness of the CheckNOW™ HIVST among Zambian adults.

**Methods:**

We conducted a cross-sectional study from 04 September 2023 – 22 September 2023 across four high-volume healthcare facilities. A total of 323 CheckNOW™ HIVST kits were distributed, with 316 consenting adults successfully enrolled in the study. Data were collected through structured questionnaires administered via face-to-face interviews following test completion, capturing information on socio-demographics, HIV testing history and user perception of the self-testing process. Descriptive statistics were employed for data analysis.

**Results:**

Among 316 participants, 56.3% (178/316) were female, and 41.5% (131/316) were aged 25–34 years. The majority (95.0%, 300/316; *p* < 0.001) found the CheckNOW™ kit easy to use, while 65.0% (206/316) had prior awareness of HIVST. Additionally, 83.6% (264/316; *p* < 0.001) followed the test instructions correctly and independently. A high proportion (98.7%, 312/316; *p* < 0.001) expressed willingness to test again, and 99.7% (315/316; *p* < 0.001) would recommend it to others.

**Conclusion:**

The CheckNOW™ blood-based HIVST kit demonstrated high usability and ease of use, supporting its potential to expand HIV testing coverage in Zambia. However, increased awareness efforts are necessary to maximise uptake and ensure broader accessibility.

**What this study adds:**

This study provides the first evidence that blood-based HIV self-testing is feasible and acceptable within Zambian clinical settings. It offers a critical new strategy to expand testing coverage and reach key populations by integrating self-testing into routine health services.

## Introduction

HIV self-testing (HIVST) has become an essential component of global strategies to increase HIV testing accessibility, particularly in resource-limited settings where healthcare infrastructure is constrained.^1^ Recognising its potential, the World Health Organization recommends HIVST as a complementary approach to conventional facility-based testing, with recent data indicating that 85% of countries had incorporated HIVST into their national HIV policies by 2023.^2,3^ Among available options, blood-based HIVST, such as the CheckNOW™ HIVST, demonstrate superior clinical performance. This rapid lateral flow immunoassay is designed for the qualitative detection of antibodies to HIV-1 and HIV-2 using a finger-prick blood sample, and has manufacturer-reported sensitivity and specificity of 99.5% and 98.5%.^4^ Studies have shown that these tests achieve 99.1% sensitivity compared to 92.3% for oral fluid tests during the critical acute infection phase.^5^ This enhanced detection capability makes blood-based tests particularly valuable for early HIV diagnosis and transmission prevention.

The effectiveness of HIVST programmes depends heavily on product usability. Recent systematic reviews highlight persistent challenges with blood-based HIVST, including difficulties with finger-prick blood collection (reported by 15% – 30% of users) and result interpretation errors that reduce testing frequency by up to 40% in some populations.^6,7,8^ These barriers are most pronounced among individuals with limited health literacy and older adults, underscoring the need for optimised test designs and clearer instructional materials.^9,10^

Zambia’s HIV epidemic reflects these global challenges while presenting unique local considerations. With an adult HIV prevalence of 11.3%,^11^ the country has made significant progress in testing coverage, yet persistent gender disparities remain: male participants represent only 35% of facility-based tests despite comparable seroprevalence rates to female participants.^11,12^ In response, Zambia’s Ministry of Health launched its 2020–2025 HIVST Scale-Up Plan, prioritising workplace and community-based distribution to reach underserved populations.^13^ This strategy builds on formative research demonstrating > 80% acceptability of HIVST across demographic groups in Zambian urban and peri-urban settings.^14,15^

This study therefore aimed at evaluating the user-friendliness, awareness, and usability of the blood-based CheckNOW™ HIVST kit in Lusaka province. By assessing real-world implementation challenges and user experiences, this study can inform the ongoing national scale-up of HIVST services and contribute to global understanding of blood-based self-testing optimisation.

## Methods

### Ethical considerations

The ethical clearance for this study was obtained from the Tropical Disease Research Centre Ethics Committee, located at the Tropical Disease Research Centre, Zambia, under reference number TDRC IRB # TDREC/095/07/23. Permission to conduct the research was granted by the Zambia National Research Authority with reference number NHRA021/10/08/2023. The principal investigator maintained active researcher registration (NHRAR-R-389/17/05/2023) throughout the study period (04 September 2023 – 22 September 2023).

Written informed consent was obtained from all participants after a detailed explanation of the study’s objectives, procedures, potential risks, and benefits. Participation was entirely voluntary, with assurances that individuals could withdraw at any time without penalty or negative repercussions. The study collected socio-demographic data from participants but did not involve the collection or analysis of bio-samples. This approach ensured ethical compliance while focusing on self-reported experiences with the HIVST kit.

To maintain confidentiality, all participant data were de-identified and securely stored, with exclusive access granted to the study team; no third-party access was permitted. The selection of participants was unbiased, enrolling only those who met the predefined inclusion criteria.

### Study design

We conducted a cross-sectional study at four high-volume health facilities in Lusaka province (04 September 2023 – 22 September 2023) to assess the user-friendliness, awareness, and usability of the CheckNOW™ blood-based HIVST kit (Abbott Rapid Diagnostic Jena [GmbH], Germany). A cross-sectional approach was chosen because it allows for a snapshot analysis of participants’ experiences, perceptions, and usability challenges at a single point in time.

Participants were randomly allocated into assisted and unassisted groups, with the assisted group receiving guidance from a trained healthcare worker or peer educator, while the unassisted group performed the self-test independently following the instructions for use. The structured study design ensured that usability factors could be compared between the two groups to assess variations in ease of use, interpretation accuracy, and willingness to recommend the test.

### Study site and population

The study was conducted in four healthcare facilities selected for their high HIV prevalence and testing volume:

- Kanyama General Hospital (Urban).
- Chawama General Hospital (Urban).
- Nangongwe Clinic (Rural).
- Chongwe District Hospital (Rural).

The study population included all adults aged ≥ 18 years who sought HIV testing at these facilities and were willing to use the CheckNOW™ HIVST kit.

### Sample size and sampling

A total of 323 CheckNOW™ HIVST kits were systematically distributed across four study facilities, with allocation proportional to each site’s average monthly testing volume. Participant recruitment occurred through consecutive random sampling during routine HIV testing services over a 14-day implementation period (04 September 2023 – 22 September 2023).

### Inclusion and exclusion criteria

#### Inclusion criteria

Adults aged ≥ 18 years, with no prior HIVST experience, seeking HIV testing, and willing to use the CheckNOW™ HIVST kit.

#### Exclusion criteria

Minors, or those declining to use self-test.

### Data collection and quality control

The study implemented rigorous quality control measures to ensure standardised data collection. The research questionnaire underwent formal validation (21 August 2023 – 25 August 2023) through a two-stage process involving content review by HIV testing experts followed by cognitive testing with 30 pilot participants. This validation process confirmed the instrument’s clarity and appropriateness for the study population.

Trained data collectors administered all study procedures following a comprehensive five-day training programme (28 August 2023 – 01 September 2023). The training curriculum covered proper HIVST administration, ethical consent procedures, and standardised data collection techniques. Only staff demonstrating full competency through practical assessments were certified to participate in the study.

Following test completion, trained data collectors conducted face-to-face interviews to collect comprehensive data using a structured electronic questionnaire. This included detailed socio-demographic characteristics (age, gender, urban-rural), clinical testing histories (previous HIV tests, results, and frequency), and qualitative user feedback about their testing experience. All physical documents, including signed consent forms and original test results, were securely stored in locked filing cabinets with restricted access limited to authorised study personnel only. All testing occurred in private designated spaces to ensure confidentiality and minimise external influences.

### Statistical analysis

Data were validated using Microsoft Excel (Version 2016, Microsoft, Redmond, Washington, United States) before being analysed in SPSS statistics V26.0 (IBM Corp., Armonk, New York, United States). Descriptive statistics summarised participant socio-demographic characteristics, HIV testing history, usability, and awareness outcomes. The Pearson’s Chi-square or Fisher’s exact tests was used to explore associations between two categorical variables, while Mann-Whitney U test was used to compare means between two groups of participants. Statistical analysis was performed using SPSS statistics V26.0 with the level of significance set at *p* < 0.05. Qualitative feedback from open-ended responses was categorised thematically, identifying trends related to ease of use, challenges, and willingness to recommend the kit.

## Results

### Participant characteristics

The study enrolled 316 participants across four Lusaka province facilities, demonstrating significant demographic variations. Gender distribution showed a statistically significant female predominance (56.3%, 178/316 vs male: 42.1%, 133/316; *p* = 0.002), with minimal missing data (1.6%, 5/316), as shown in Table 1. Age groups differed significantly from national census distributions (*p* < 0.001), with 25–34-year-olds representing the largest cohort (131/316, 41.5%), followed by 18–24-year-olds (35.7%, 113/316) and ≥ 35-year-olds (22.8%, 72/316). Urban residents were over-represented (60.1%, 190/316 vs rural: 39.9%, 126/316; *p* < 0.001). Testing history revealed exceptionally high prior engagement (95.6%, 302/316; *p* < 0.001 versus the national rate of 89%), with most recent tests occurring ≤ 1 year prior (61.9%, 187/302; *p* < 0.001), demonstrating frequent engagement with testing services. All four facilities demonstrated high testing uptake (97.8%, 316/323). Site-specific distribution patterns are shown in Table 2.

**TABLE 1:** Participant socio-demographic characteristics, Lusaka province, Zambia, 04 September 2023 – 22 September 2023.

Variable	Category	*n*	%	*p*-value
Gender		-	-	0.002*
	Female	178	56.3	-
	Male	133	42.1	-
	Missing	5	1.6	-
Age group (years)		-	-	0.001*
	18–24	113	35.7	-
	25–34	131	41.5	-
	≥ 35	72	22.8	-
Residence		-	-	< 0.001*
	Urban	190	60.1	-
	Rural	126	39.9	-
HIV testing history		-	-	< 0.001*
	Previously tested	302	95.6	-
	First-time testers	14	4.4	-
Time since last test		-	-	< 0.001*
	≤ 1 year	187	61.9	-
	> 1 year	84	27.8	-
	Missing	31	10.2	-

* , *p*-values were obtained using the Chi-square test and a *p*-value < 0.05 was considered statistically significant.

**TABLE 2:** Kit utilisation by study site, Lusaka province, Zambia, 04 September 2023 – 22 September 2023.

Facility	Distributed (*n*)	Used (*n*)	Utilisation rate (%)
Kanyama General Hospital	87	80	92.0
Chongwe District Hospital	66	66	100.0
Chawama General Hospital	110	110	100.0
Nangongwe Clinic	60	60	100.0

**Total**	**323**	**316**	**97.8**

### User-friendliness and awareness

The CheckNOW™ HIVST kit exhibited outstanding usability and acceptability in the study. As shown in Table 3, 95.0% (300/316; *p* < 0.001) of participants found the test easy to use, with only 4.4% (14/316) reporting difficulties. Prior awareness of HIVST varied significantly (65.0%, 206/316; *p* < 0.001), and among those familiar with self-testing, 58.3% (120/206) had prior testing experience. Notably, a pronounced gender disparity was observed among previous users (*p* < 0.001): 69.2% were female participants (83/120), 29.2% were male participants (35/120), and gender information was unavailable for 1.6% (2/120). The kit demonstrated near-universal acceptability, with 98.7% (312/316; *p* < 0.001) of participants willing to reuse it and 99.7% (315/316; *p* < 0.001) recommending it to others, underscoring its potential for widespread adoption.

**TABLE 3:** Usability and awareness outcomes, Lusaka province, Zambia, 04 September 2023 – 22 September 2023.

Assessment category	Response	*n*	%	*p*-value
Perceived ease of use		-	-	< 0.001*
	Yes	300	95.0	-
	No	14	4.4	-
	Missing	2	0.6	-
Prior HIVST awareness		-	-	< 0.001*
	Aware	206	65.0	-
	Unaware	104	33.0	-
	Missing	6	2.0	-
Among aware participants		-	-	< 0.001*
	Previous users	120	58.3	-
	First-time users	73	35.4	-
	Missing	13	6.3	-
Gender distribution of prior users		-	-	< 0.001*
	Female	83	69.2	-
	Male	35	29.2	-
	Missing	2	1.6	-
Future use intentions		-	-	< 0.001*
	Willing	312	98.7	-
	Unwilling	3	1.0	-
	Missing	1	0.3	-
Recommendation likelihood		-	-	< 0.001*
	Yes	315	99.7	-
	No	1	0.3	-

HIVST, HIV self-testing.

* , *p*-values < 0.001 were considered statistically significant.

### User experience with CheckNOW™ HIV self-test kit

**FIGURE 1:**
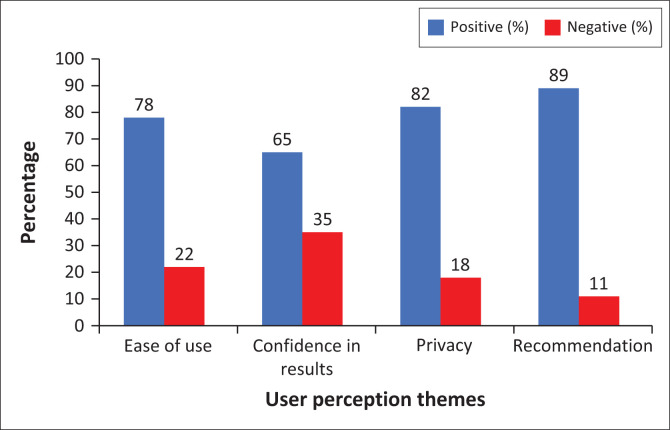
User experience themes with CheckNOW™ HIV self-testing kit, Lusaka province, Zambia, 04 September 2023 – 22 September 2023.

Figure 1 indicates participants’ experiences with the CheckNOW™ HIVST kit. Positive feedback includes responses indicating ease of use, confidence in result interpretation, satisfaction with privacy, and willingness to use or recommend the kit. Negative feedback includes reports of difficulty in blood collection, challenges with self-pricking, uncertainty in result interpretation, or preference for assisted testing. Over three-quarters (78%, 246/316) of respondents described the test as simple, though 22% (70/316) noted challenges with blood collection. More than half (65%, 205/316) expressed high trust in the accuracy of their results, while 35% (111/316) desired clearer instructions for interpretation. The majority (82%, 259/316) valued the discretion of self-testing, with 18% (57/316) suggesting improvements in packaging for enhanced confidentiality; 89% (281/316) would endorse the kit to others, citing convenience and reliability.

### Usability of the CheckNOW™ HIV self-test

The CheckNOW™ HIVST kit demonstrated excellent performance in our evaluation, with statistically significant findings, as shown in Table 4. Participants showed significantly higher rates of unassisted administration (58.9%, 186/316) compared to assisted use (23.7%, 75/316; *p* < 0.001), indicating successful independent implementation. The accuracy of participants’ interpretation of the test results was exceptionally high at 97.5% (308/316; *p* < 0.001), with only 1.6% (5/316) errors. This was assessed by comparing each participant’s recorded result against a trained healthcare worker’s independent reading of the same used test device. While 83.6% (264/316) of users comprehended instructions independently, the 16.1% (51/316) requiring assistance remained statistically significant (*p* < 0.001), highlighting a need for enhanced instructional materials. Test results followed expected distributions, with 90.8% negative (287/316), 7.9% positive (25/316), and no invalid results. These outcomes, combined with minimal missing data and perfect test validity, confirm the kit’s robust real-world performance while identifying key opportunities for optimising user support through refined instructional aids for the 16.1% needing assistance (*p* < 0.001), and targeted training for healthcare workers supporting the 23.7% assisted cases.

**TABLE 4:** HIV self-testing performance metrics, Lusaka province, Zambia, 04 September 2023 – 22 September 2023.

Assessment category	Result	*n*	%	*p*-value
Administration method		-	-	< 0.001*
	Unassisted	186	58.9	-
	Assisted	75	23.7	-
	Missing	55	17.4	-
Test results		-	-	0.372
	Negative	287	90.8	-
	Positive	25	7.9	-
	Invalid	0	0.0	-
	Missing	4	1.3	-
Result interpretation		-	-	< 0.001*
	Correct	308	97.5	-
	Incorrect	5	1.6	-
	Missing	3	0.9	-
IFU comprehension		-	-	< 0.001*
	Independent	264	83.6	-
	Needed help	51	16.1	-
	Missing	1	0.3	-

IFU, instructions for use.

* , *p*-values were obtained using the Chi-square test and a *p*-value < 0.05 was considered statistically significant.

## Discussion

Our evaluation of the CheckNOW™ blood-based HIVST kit in Zambia revealed several key findings with important implications for HIV testing programmes. The exceptionally high acceptability rates (95.0% ease of use, 98.7% willingness to reuse, and 99.7% recommendation rate) demonstrate the potential of well-designed blood-based HIVST kits to expand testing access. These results align with recent multi-country studies showing that optimised HIVST kits can increase testing uptake by 40% – 60% among hard-to-reach populations, including men and adolescents.^16,17,18^ The near-universal recommendation rate is particularly noteworthy, as peer-driven distribution has been shown to increase first-time testing threefold in similar settings.^19,20^

The gender distribution of our participants (56.3% female) reflects a well-documented pattern across sub-Saharan Africa, where women are consistently 1.8 times more likely to utilise HIVST services than men.^21^ This disparity stems from multiple factors, including men’s greater occupational mobility (particularly in mining and transportation sectors), and perceptions that clinical settings are ‘female spaces’.^22,23^ Successful interventions from Uganda’s male-friendly HIVST programme, which increased testing rates among men by 35% through workplace distribution, could be adapted for Zambia’s context.^24,25^ Targeted outreach to male-dominated workplaces and social venues should be prioritised in scale-up plans.

The 97.5% success rate in performing all testing steps correctly suggests that previous concerns about the complexity of blood-based HIVST may be overcome through improved kit design. Key features contributing to this success likely include: intuitive pictorial instructions (shown to improve accuracy by 22% in low-literacy populations),^26^ pre-filled reagents that minimise procedural steps,^27^ and clear visual indicators for result interpretation.^28^ However, our findings contrast with studies showing 15% lower success rates among older adults in Tanzania,^29^ highlighting the need for age-specific usability assessments during implementation.

The 65% pre-test awareness level we observed, while comparable to rates in South Africa (69.9%),^30^ indicates substantial room for improvement. Zambia could adapt successful awareness strategies from other settings, including the use of community-based ‘HIVST champions’ (shown to increase awareness to 81% in pilot programmes^31^), integration with mobile health platforms for results interpretation support,^32^ and targeted messaging through male-oriented community networks.^33^

### Study limitations

While this study provides valuable insights into HIVST usability, three key limitations should be considered. First, the single-site urban design may limit generalisability to rural populations where national literacy rates are 37% lower than urban populations. Second, reliance on self-reported data introduces potential social desirability bias, suggesting future research should incorporate direct observational methods for more objective assessment. Finally, the cross-sectional design cannot evaluate critical longitudinal outcomes including sustained testing frequency over time, linkage-to-care rates following self-testing, or patterns of repeat test utilisation. These limitations highlight the need for multi-site longitudinal studies that combine usability assessments with clinical outcome tracking in diverse population groups.

### Conclusion and recommendations

The CheckNOW™ HIVST kit’s strong performance, evidenced by its 95.0% usability rate and 99.7% recommendation score, supports its inclusion in the national HIV testing strategy in Zambia. To maximise impact, we recommend: gender-sensitive distribution through workplaces and male-oriented community venues, and targeted awareness campaigns addressing persistent knowledge gaps. These findings contribute to growing evidence that well-designed blood-based HIVST can play a crucial role in achieving universal testing targets, particularly among populations that face barriers to facility-based testing.
